# Rupture of solidified ancient magma that impeded preceding swarm migrations led to the 2024 Noto earthquake

**DOI:** 10.1126/sciadv.adv5938

**Published:** 2025-10-15

**Authors:** Ryota Takagi, Keisuke Yoshida, Tomomi Okada

**Affiliations:** Research Center for Prediction of Earthquakes and Volcanic Eruptions, Graduate School of Science, Tohoku University, Sendai 980-8578, Japan.

## Abstract

An intense earthquake swarm lasting ~3 years ultimately led to the 2024 *M*_w_ (moment magnitude) 7.5 earthquake in the Noto Peninsula, Japan. The spatial complexities in swarm evolution and earthquake rupture have been observed, but the factors controlling these complexities remain unclear. Using high-resolution subsurface imaging with dense seismic observation, we identified a high-velocity body collocated with the major slip zone, which the preceding swarm avoided. The spatial distribution and absolute velocity of the high-velocity body and the adjacent ring-shaped swarm cluster indicate that the high-velocity body is a solidified ancient magma. It initially acted as an impermeable barrier to the fluid migrations that triggered swarm earthquakes, eventually rupturing as an asperity of the 2024 earthquake. Our observation suggests that the heterogeneity in fault zone permeability, originating from ancient volcanic activity (>15 million years ago), controlled the present-day swarm evolution and the large earthquake generation.

## Teaser

Solidified ancient magma acted as a barrier to precursor fluid migrations and then as the core of the 2024 Noto earthquake.

## INTRODUCTION

**Fig. 1.**
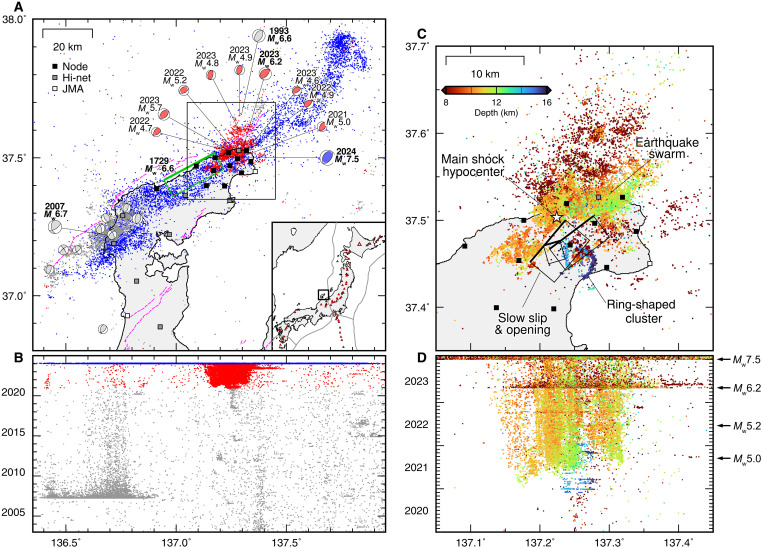
Spatiotemporal distributions of crustal earthquakes in the Noto Peninsula, Japan. (**A**) Crustal earthquake distributions of the JMA catalog (*M* ≥ 2) for three periods: 1 January 2003 to 30 November 2020 (gray), 1 December 2020 to 31 December 2023 (red), and 1 January 2024 to 31 January 2024 (blue). The squares are the station locations (black: seismic nodes; gray: Hi-net, white: JMA). The focal mechanism of the 1993 earthquake is from the global centroid moment tensor catalog (*51*), while other focal mechanisms (*M*_w_ ≥ 4.5) are from the NIDE F-net catalog (*52*). The green rectangle and pink curves are the 1729 earthquake (*1*) and active faults (*53*). The red triangles in the inset map indicate Quaternary volcanoes. (**B**) Space-time plot of the JMA hypocenter catalog (*M* ≥ 1). (**C**) Relocated 2020–2023 swarm hypocenters (*17*) and the fault models of the slow slip and opening coinciding with the earthquake swarm (*11*). The hypocenters are color coded by the hypocenter depth, corrected to be 1.5 km shallower than the original values to account for systematic shifts because of the assumed 1D velocity structure for the hypocenter location (*12*). The white star is the *M*_w_ 7.5 hypocenter. (**D**) Space-time plot of the relocated swarm hypocenters.

The Noto Peninsula region, Japan, has experienced large earthquakes and ancient volcanic activity. Damaging crustal earthquakes have occurred in the past in this region including the 1729 moment magnitude (*M*_w_) ~ 6.6 (*1*), 1993 *M*_w_ 6.6, and 2007 *M*_w_ 6.7 earthquakes (Fig. 1A). The *M*_w_ 7.5 Noto earthquake on 1 January 2024 was much larger than these events and ruptured an area of ~150 km in length (*2*, *3*). This event uplifted the northern coast by ~4 m, and marine terraces along the northern coast infer the recurrence of such substantial uplift (*4*, *5*). There are no Quaternary volcanoes in the Noto Peninsula; however, active volcanic activity occurred during the back-arc rifting and opening of the Japan Sea at ~15 to 30 million years ago (Ma) (*6*).

Notably, a fluid-driven earthquake swarm occurred near the hypocenter of the 2024 Noto earthquake as a precursor. The earthquake swarm, which began in August 2018, became active in December 2020, 3 years before the *M*_w_ 7.5 earthquake (Fig. 1). The upward migration of swarm hypocenters (*7*–*10*), geodetically observed aseismic slow slip and crack opening (*11*), low seismic velocity anomalies (*12*, *13*), an *S*-wave reflector (*9*), and low-resistivity bodies (*14*) indicated that crustal fluids triggered the earthquake swarm. The hypocenter distribution shows a local ring-shaped cluster, seismicity characteristics frequently observed in volcanic areas (*15*), implying that the earthquake swarm was hosted by heterogeneous structures related to the ancient volcanic activity (*9*).

Detailed kinematic rupture modeling and hypocenter distributions revealed the complex rupture in the initial stages of the *M*_w_ 7.5 earthquake (*3*, *16*, *17*). However, it remains unclear what controls the complex spatial variability of seismicity and rupture patterns. Here, we estimated a detailed three-dimensional (3D) *S*-wave velocity structure beneath the Noto Peninsula on the basis of dense seismic observations. With the high-resolution *S*-wave velocity structure, we revealed a contrast in *S*-wave velocity between the inside and outside of the swarm area and a distinct high-velocity body collocated with the large slip zone of the *M*_w_ 7.5 earthquake. We infer the origin of the high-velocity body and its roles on the swarm evolution and the generation of the 2024 earthquake.

## RESULTS

### High-resolution subsurface structure

High-resolution seismic imaging relies primarily on dense seismic observations. The seismic stations of the permanent seismic network have a spacing of ~20 km (*12*, *18*); a detailed study of the relationship between the seismic velocity structure, earthquake swarm, and the *M*_w_ 7.5 earthquake is challenging. To overcome this difficulty and achieve high-resolution imaging, we installed 12 seismic nodes (IGU-BD3C-5, SmartSolo Inc.) on the Noto Peninsula with an average separation distance of 5 km (Fig. 1A). We also used eight Hi-net stations (*19*) from the National Research Institute for Earth Science and Disaster Resilience (NIED) and two short-period seismic stations from the Japan Meteorological Agency (JMA). The observational data covered two periods in 2023: 14 October to 1 November and 16 November to 28 November, with a total length of 32 days, 1 month before the 2024 *M*_w_ 7.5 earthquake.

Ocean wave–generated seismic surface waves, known as microseisms and ambient seismic noise, were used to image the subsurface structure. Because microseisms are continuously generated by pressure fluctuations caused by ocean waves, they appear as random noise in the observed waveforms. However, the long-term average of the cross-correlation function between two stations emerges as the interstation Green’s function, which describes the wave propagation (*20*). The seismic nodes exhibit high-quality data of microseisms, and the averaged interstation cross-correlation functions of continuous seismic records over 32 days show clear surface wave propagations of Rayleigh and Love waves at ~0.05 to 0.5 Hz. Considering the lower horizontal resolution at lower frequencies, primarily due to long surface wave wavelengths and the limited number of interstation phase velocity measurements, we used frequencies above 0.1 Hz to estimate the 3D velocity structure (Materials and Methods). This frequency range allows us to constrain the *S*-wave velocity down to a depth of ~15 km.

**Fig. 2.**
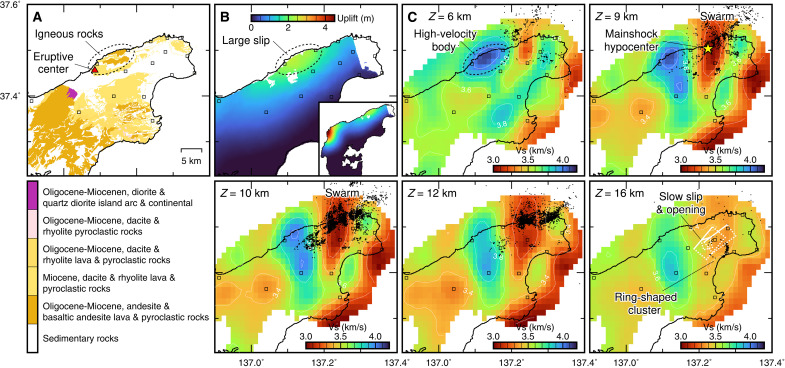
Geology, coseismic uplift, and *S*-wave velocity structure. (**A**) Geological map showing igneous rock distribution (*21*). The red triangle is the eruptive center of the Miocene dacite and rhyolite rocks (*22*). The squares show the seismic stations used in the present study. (**B**) Coseismic uplift as a result of the 2024 *M*_w_ 7.5 Noto Peninsula earthquake (*3*). (**C**) Horizontal cross sections of the estimated *S*-wave velocity model. The white rectangles show the source faults of slow slip and opening that coincide with the earthquake swarm (*11*). The dots indicate swarm earthquakes from December 2020 to December 2023 within ±0.5 km from the slice depths. The yellow star represents the *M*_w_ 7.5 hypocenter. The hypocenter depths are shifted shallower by 1.5 km.

**Fig. 3.**
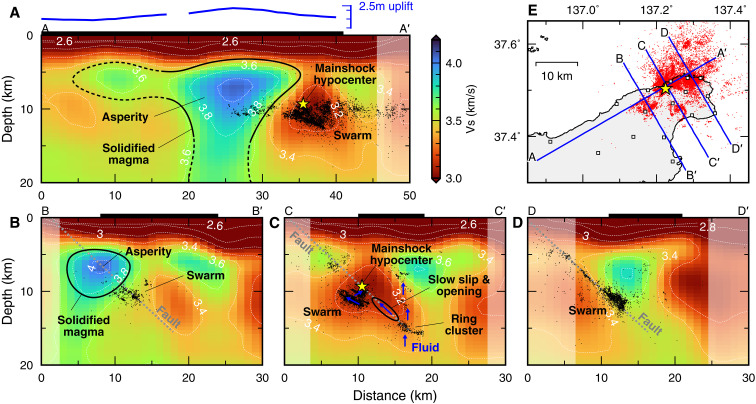
Vertical cross sections of the estimated *S*-wave velocity model with interpretation. (**A**) Vertical cross sections along the southwest-northeast profile shown in (**E**). The blue curve shows the coseismic uplift along the profile (*3*). The shaded areas were not resolved in the present study. The black dots and yellow triangle represent the hypocenters of swarm earthquakes from December 2020 to December 2023 and the 2024 *M*_w_ 7.5 earthquake (*17*). The horizontal thick black bar represents the land area. (**B** to **D**) Vertical cross sections along the northwest-southeast profiles shown in (E). The gray dashed line represents the source fault inferred from swarm hypocenters (*17*).

The extracted surface waves were then used to image a 3D *S*-wave structure via seismic tomography (Materials and Methods), and the *S*-wave structure was compared with precisely determined hypocenters (*17*). The results showed that the Noto Peninsula has a very complex crustal structure around the earthquake swarm (Figs. 2 and 3). The distribution of swarm earthquakes correlated well with structural anomalies. Most swarm earthquakes occurred at depths of 9 to 12 km within the low-velocity regions (*Z* = 9 to 12 km in Fig. 2C). Swarm earthquakes are distributed in distinct clusters, avoiding high-velocity regions, which is evident at a depth of 10 km. The deep ring-shaped cluster around the 16-km depth and the sources of slow slip and opening are also located in the area where *S*-wave velocity is relatively low (*Z* = 16 km in Fig. 2C). Moreover, the hypocenter of the 2024 *M*_w_ 7.5 earthquake is located within the low-velocity swarm area.

The most notable feature is the high-velocity body located in the region immediately west of the swarm area (Figs. 2C and 3A). This high-velocity body is continuously distributed from a ~20- to 5-km depth, and it is localized around the northern coast of the Noto Peninsula in the shallow depth (Fig. 2C). The surface projection of the high-velocity body at a depth of 6 km is collocated with Oligocene-Miocene igneous rocks (*21*) and the substantial uplift region because of the 2024 *M*_w_ 7.5 earthquake (Figs. 2 and 3A) (*3*). The velocity contrast between the swarm area and its adjacent uplift area is also evident from phase velocity measurements and synthetic recovery tests (Materials and Methods).

## DISCUSSION

### Role of the solidified magma

There are three pieces of evidence suggesting that the high-velocity body originates from solidified ancient magma. First, the distribution of the high-velocity body at a ~5- to 10-km depth spatially correlates with the surface Oligocene-Miocene igneous rocks. Andesitic and basaltic-andesite rocks at ~25 to 28 Ma are distributed where the high-velocity body is localized at the northern coast, and the eruptive center of the latest dacitic and rhyolitic rocks at ~15.6 Ma is located within the high-velocity region (*22*). Second, the absolute velocity of the high-velocity body ranging from ~3.8 to 4.0 km/s is consistent with laboratory measurements of mafic intrusive rocks (*23*). Mafic intrusion is a common feature in rift volcanisms (*24*). Third, the southern swarm cluster shows ring-shaped hypocenter distributions. A previous study reported seismic activities on ring faults that formed associated with calderas in volcanic areas (*15*). Although the depth of the hypocenters of this ring-shaped swarm cluster is too deep for a direct link to past calderas, similar ring-shaped structures can be formed within the upper and middle crust because of magma intrusions (*25*). This ring-shaped activity may be related to such magmatic structures (*9*). These multiple lines of evidence indicate that the intruded mafic magma, which served as the source of the rift volcanic activity, has cooled and solidified, likely manifesting as the current distinct high-velocity body. In the solidified magma, normal faults might have been formed during the later stages of the back-arc opening, or reverse faults could have been created by the subsequent compressive stress field, and the 2024 Noto Peninsula earthquake may have ruptured such faults.

The high-velocity body is collocated with the coseismic uplift area because of the 2024 *M*_w_ 7.5 earthquake (Fig. 2, B and C). The large uplift is the direct evidence of large slip that occurred in this area (*3*). The spatial coincidence of the high-velocity body and the uplift area suggests that the solidified magma acted as a major asperity ruptured by the 2024 *M*_w_ 7.5 earthquake. The hypocenter of the 2024 *M*_w_ 7.5 earthquake is located within the low-velocity region east of the high-velocity body, and the initial-stage rupture started outside the high-velocity asperity. This asperity possibly slowed down the initial-stage rupture, and the failure of this asperity allowed this event to grow by causing dynamic ruptures to propagate further west and east (*16*). In contrast to the *M*_w_ 7.5 rupture, the preceding swarm occurred intensively near the southern and eastern edges of the high-velocity body but did not migrate into it. The migrations of the earthquake swarm provide evidence for diffusive fluid flow driving swarm activity (*7*), and the fluid diffusivity primarily reflects the permeability of the medium. The lack of the swarm within the high-velocity body shows that the solidified magma acted as an impermeable barrier for the fluid migrations before 2024.

**Fig. 4.**
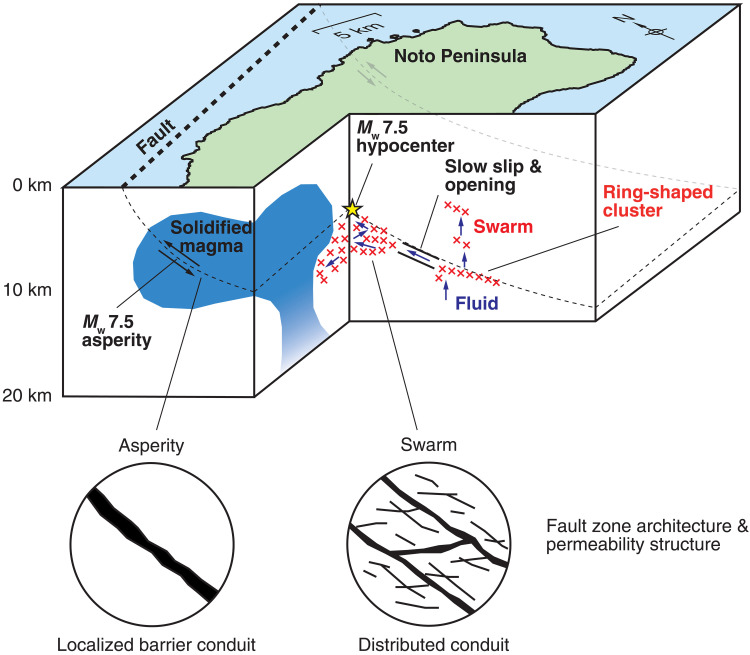
Schematic illustration of the heterogeneous structure controlling the swarm and the large earthquake beneath the Noto Peninsula.

Spatiotemporal variations in the permeability affect the genesis of crustal earthquakes. The fault-valve model defines cyclic fault failure controlled by both stress and fluid pressure (*26*). The increase in fluid pressure during the seismic cycle is maintained by the permeability barrier at the root of the fault zone and the continuous fluid supply from the deeper part of the crust. The permeability barrier may be formed by a clay-rich or cataclastic gouge and/or hydrothermal precipitation and cementation (*26*), and the sealing process is controlled by the fault zone structure. Our results suggest that the fault zone formed in the solidified magma acted as a large impermeable barrier before the 2024 Noto earthquake, whereas the fault zone permeability is relatively high in the swarm area (Fig. 4). A plausible explanation of the origin of the permeability contrast is the fault zone architecture depending on the lithology and physical properties of the host rock (*27*). The fault zone in the rigid solidified magma may tend to be localized because of compliance contrast between the fault zone and the host rock, and the localized fault zone acts as an impermeable barrier before large earthquakes and as a localized conduit after (Fig. 4). The localized fluid flow after large earthquakes may facilitate fault healing and sealing through hydrothermal precipitation (*28*). In contrast, fault and fracture networks may be developed in the less rigid surrounding rocks, which serve as distributed fluid conduits and host the earthquake swarm. The present model can also be interpreted as reflecting the deformation concentration and ease of fault zone maturation, which depend on the host rock property. Previous studies reported that mature faults with a localized damage zone tend to have high rupture velocity and host large events and immature faults with a distributed damage zone have slow rupture velocity and host small events (*29*, *30*). The asperity of the *M*_w_ 7.5 earthquake may correspond to a mature fault within the rigid solidified magma. Conversely, the preceding swarm earthquakes and the initial slow rupture of the *M*_w_ 7.5 earthquake may be hosted by immature faults within less rigid surrounding rocks.

Earthquake swarms have consistently raised concerns regarding whether they will lead to major damaging earthquakes, while many earthquake swarms ended without causing a major earthquake (*31*–*33*). This study shows that permeability heterogeneity around swarm areas is a critical factor controlling the swarm evolution and large earthquake generations and seismic imaging with dense observations potentially reveals the spatial variations in permeability. Comparing the heterogeneous permeability structure around the earthquake swarms that caused a major earthquake with those that did not can enhance our understanding of the physics governing earthquake swarm evolution and cessation and also can contribute to seismic hazard assessment in swarm-prone areas.

## MATERIALS AND METHODS

### Ambient noise cross-correlation functions

High-quality data in the low-frequency range around primary and secondary microseisms are crucial for ambient noise surface wave tomography to estimate crustal structures related to seismogenesis. The seismic nodes clearly show secondary microseisms (fig. S1). At frequencies lower than ~0.2 Hz, the sensor noise power is approximately a few times larger than that of the Hi-net velocity sensor with a natural frequency of 1 Hz. Both the seismic node and Hi-net stations had instrument noise greater than that of the primary microseisms observed by the F-net broadband station below ~0.1 Hz. Note that the relatively higher noise levels of the nodal and Hi-net stations at frequencies below 0.2 and 0.1 Hz, respectively, do not indicate directly their frequency limits. This is because the noise levels primarily reflect uncorrelated instrument noise among stations, and the long-term averaging of interstation cross-correlation functions allows the surface wave signals to overcome this uncorrelated instrument noise (*34*).

We computed the averaged interstation cross-correlation functions of continuous seismic records over 32 days for the pairs of vertical (Z), radial (R), and transverse (T) components after removing time windows that included anomalous amplitudes. The cross-correlation functions of ambient seismic noise show clear Rayleigh and Love wave propagations at 0.05 to 0.5 Hz (fig. S2A), whereas faster *P* waves are evident on the ZZ and RR components at 0.1 to 0.25 Hz, which can be attributed to teleseismic *P*-wave microseisms (*35*). Taking the difference between the ZR and RZ components successfully eliminated the *P* waves and enhance surface wave signals (*36*). Although the array-based dispersion spectra indicate that the cross-correlation functions extract surface wave signals below 0.05 to 0.1 Hz (fig. S2C), we used the frequency range at 0.1 to 0.45 Hz for estimating the 3D velocity structure considering the horizontal resolution and surface wave wavelengths, as described in the following section. Surface waves are primarily sensitive to *S*-wave velocity structures, and the sensitivity kernels indicate that our surface wave inversions can constrain the *S*-wave velocity down to a depth of ~15 km (fig. S2B).

### Ambient noise surface wave tomography

The extracted surface waves were used to estimate the 3D *S*-wave velocity structure via ambient noise surface wave tomography. The ambient noise surface wave tomography consists of four steps (*37*): (i) array-based estimation of reference phase velocity dispersion, (ii) measurement of phase velocity anomaly at each station pair, (iii) 2D phase velocity tomography at each frequency, and (iv) construction of a 3D velocity model from a series of local 1D inversions at all the grid points.

#### First step: Reference phase velocity measurement

The first and second steps follow the model-space waveform fitting method (*38*). The multimode dispersion curves were estimated by fitting the theoretical cross-spectra to the observed spectra. This method uses a 1D *S*-wave velocity structure as a model parameter for measuring the phase velocity dispersion curves. The advantages of this method include the simultaneous use of multicomponent cross-correlation functions and robust dispersion measurements owing to the physical constraints of 1D structures. We fitted the theoretical cross-spectra to all ZZ, RR, TT, and ZR-RZ components simultaneously, where ZZ, RR, TT, and ZR-RZ indicate the vertical-vertical, radial-radial, transverse-transverse, and the difference between vertical-radial and radial-vertical components, respectively.

In the first step, we estimated the reference phase velocity by array-based cross-spectral fitting. The frequency ranges for the spectral fitting were set at 0.05 to 0.45 Hz for the fundamental-model Rayleigh and Love waves and 0.25 to 0.45 Hz for the first-overtone Rayleigh wave. The first-overtone Rayleigh wave was included in the cross-spectral fitting during the first and second steps to reduce a possible bias of the fundamental-mode measurements because of the first-overtone signals (fig. S2C). In the subsequent third and fourth steps, the first-overtone Rayleigh wave was excluded from subsurface structure estimation because of its small amplitude and low measurement accuracy. In the frequency range lower than ~0.25 Hz, the first-overtone dispersion curve does not fit the data, which may be due to leaking mode energy (*39*). In addition, teleseismic *P* waves are evident in the ZZ and RR components around 0.2 Hz. Because the cross-spectra include these unmodeled phases, we used the residuals of the array-based cross-spectral fitting as the weighting factors to reduce the contributions of such components and frequencies in the second-step pairwise cross-spectral fitting (*38*). Sixteen cubic B-spline functions were used to represent the 1D model structure (fig. S3). We arranged denser knots in the shallow part to represent a sharp velocity gradient in the shallow crust and a wide range to search for the *S*-wave velocity structure. We estimated 100 reference 1D velocity models and dispersion curves by randomly resampling pairs of stations within the seismic array (fig. S2D).

#### Second step: Pairwise phase velocity measurement

In the second step, the phase velocity perturbation for each path is estimated relative to the reference dispersion curves. From the surface to the bottom of the 1D model, the search ranges were set at ±50% from the reference values for the coefficients of the first three basis functions, ±30% for the next eight basis functions, and ±10% for the next four basis functions, and the coefficient of the bottom basis function was fixed as the reference value, considering the depth sensitivity of the surface waves. We estimated 100 measurements of the phase velocity perturbations from the 100 reference models and used their mean and standard deviation as the input for the third-step phase velocity tomography.

We performed quality control on phase velocity measurements for each station pair using multiple criteria: interstation distance, variance reduction, estimation error, and outlier detection. First, we only used measurements with interstation distances longer than half a wavelength. There is no low-frequency limit in principle because we fit the theoretical cross-spectra in the frequency domain and do not use the far-field approximation as in time-domain measurements. However, we applied the half-wavelength criterion to stabilize the measurements, considering the first zero (~0.38 wavelength) of the Bessel function that represents the theoretical cross-spectra. Next, we used the variance reduction when fitting the cross-spectra (*38*). The variance reduction was calculated within a bandwidth of ±0.05 Hz from each frequency. We computed the variance reduction for each component and the variance reduction contribution of each mode (fig. S4) (*38*). We set criteria that the former must be at least 15% and the latter must be at least 10% for each mode. For Rayleigh waves, we only used measurements where at least one of the four components (ZZ, ZR-RZ, RR, and TT) met the criteria. For Love waves, we applied the same criteria to at least one of the two components (RR and TT). Third, we removed data where the measurement error, estimated from the bootstrap method, exceeded 0.2 km/s. Last, we removed outliers using the dispersion curves from all station pairs. Given that the influence of phase velocity heterogeneity is averaged out for station pairs with larger interstation distances, we arranged the phase velocity measurements in order of interstation distance. We then calculated the mean and standard deviation of 30 phase velocity measurements centered on the target measurement and removed the target data if they exceeded three times the standard deviation from the mean. The final dispersion data and the number of data are shown in fig. S5. The estimated pairwise phase velocities showed spatial variations, indicating a low-velocity area in the earthquake swarm area (figs. S6A and S7A).

#### Third step: Phase velocity tomography

In the third step, the 2D phase velocity map for each surface wave mode at each frequency is estimated. This involved a two-step approach. The first step is based on the ray approximation using the fast-marching surface wave tomography package (*40*), and the second step incorporates the finite-frequency effect based on the Rytov approximation with asymptotic Green’s function of surface waves (*41*). The results of the first-step ray-based tomography (figs. S6B and S7B) are updated with the second-step finite-frequency kernels (figs. S6C and S7C) to obtain the phase velocity maps, which incorporate the finite-frequency effect (figs. S6D and S7D) (*42*). The finite-frequency kernels were calculated as the average within ±0.025 Hz from the center frequency. We used a grid spacing of 0.025° in both the longitudinal and latitudinal directions. Initial phase velocity maps were constructed using the 3D *S*-wave velocity model estimated by ambient-noise surface wave tomography using Hi-net data in the 0.05- to 0.2-Hz range (*43*). We adopted second-derivative smoothing and damping to regularize the inversion. Both the smoothing and damping parameters were set to 10, which minimized the Akaike Bayesian information criterion (*44*, *45*) for the Rayleigh wave at 0.1 Hz in the ray-based tomography.

The misfit values were reduced after the two-step phase velocity tomography (figs. S6G and S7G). The estimated phase velocity maps show a low-velocity area in the northeastern Noto Peninsula and a high-velocity area west of the low-velocity area at frequencies above ~0.1 Hz (figs. S6D and S7D). The diagonal components of the posterior covariance and resolution matrices indicate that the phase velocity maps in the frequency above ~0.1 Hz are well resolved in the northeastern Noto Peninsula owing to dense seismic node observations. In the next-step depth inversion, we used phase velocities with a diagonal component of the resolution matrix larger than 0.05 considering the ray coverage (figs. S6F and S7F). The frequency ranges and the number of phase velocities used in the next step are shown in fig. S8. The standard deviations of phase velocities were used as the data uncertainty in the next 1D inversions (figs. S6E and S7E).

#### Fourth step: 3D model construction by local 1D inversions

Fourth, a 3D *S*-wave velocity model was constructed by conducting local 1D inversions at each grid point (fig. S9). Local dispersion curves at each grid point extracted from the phase velocity maps were used to estimate the 1D *S*-wave velocity structure using iterative linearized inversion (*46*, *47*). Sensitivity kernels were computed using the DISPER80 package (*48*). We represented the 1D model using 60 layers, with each layer having a thickness of 1 km. The topography was corrected by increasing the thickness of the shallowest layer with elevation. Only the *S*-wave velocity structure was used as a model parameter, and the *P* wave, density, and sensitivity kernel were scaled using empirical formulas (*49*). The same 1D velocity model explains both Rayleigh and Love waves well (fig. S9), indicating that the radial anisotropy is not strong. Thus, we estimated the isotropic *S*-wave velocity using both Rayleigh and Love waves simultaneously. We used the 3D *S*-wave velocity model (*43*) as the initial 3D model, which is the same as the model used for computing the initial values for 2D phase velocity tomography. Even if we use a single 1D velocity model as the initial model for all grid points and if we invert Rayleigh and Love waves separately, we can image the velocity anomaly discussed (fig. S10), which indicates the robustness of our results and interpretation. The misfit values of the phase velocity data are lower than ~0.1 km/s in the entire region (fig. S8A).

We used a Gaussian prior model covariance matrix for the smoothness constraint (*47*) and selected its correlation length of 2.5 km and standard deviation of 0.2 km/s. Although the choice of these parameters was arbitrary, we confirmed that changing them did not affect our conclusions. With a longer correlation length and small standard deviation, the modeled dispersion curves do not fit the phase velocity data well, and a large standard deviation with no vertical correlation causes the 1D velocity model to oscillate unstably (fig. S11). In addition, a similar inversion using longer-period teleseismic surface waves adopted a correlation length of 5 km and standard deviation of 0.1 km/s for the crust (*42*). Because our ambient noise data in a shorter period were sensitive to a shallower structure than the teleseismic data (*42*), the choice of parameters was within a reasonable range. The initial 3D model (*43*) is not sensitive to the shallow low-velocity layer because of the 0.02- to 0.2-Hz frequency range used. Thus, a larger standard deviation of 1 km/s was chosen for the shallowest layer.

#### Model validation and synthetic tests

The velocity contrast between the swarm area and the adjacent large slip area is also evident from the array-based phase velocity measurements and pairwise phase velocity measurements (fig. S12). We used the ZR-RZ component data within subarrays in the two areas. The Rayleigh wave phase velocity in the large slip area is higher than that in the swarm area (fig. S12, A and B). The phase velocity dispersion curves for the two individual station pairs along the northern coast also indicate higher phase velocities in the large slip area within the frequency range of 0.1 to 0.25 Hz (fig. S12, D and E), which is sensitive to the upper crust (fig. S12F). As both station pairs are almost parallel to each other, the phase velocity contrast cannot be attributed to nonisotropic distributions of the ambient noise source.

The possible bias as a result of nonisotropic source distributions tends to be larger in shorter interstation distances (*50*). To check for the potential bias, we tested this by increasing the threshold value of the interstation distances for the pairwise phase velocity measurements from half a wavelength to one wavelength. While a one-wavelength criterion results in a loss of horizontal resolution because of the exclusion of short-distance station pairs, the high velocity anomalies in the large-slip region and the low velocity anomalies in the earthquake swarm region persist (fig. S10D). This result, combined with the phase velocity measurements from the two parallel station pairs mentioned previously (fig. S12, D and E), indicates that biases from nonisotropic noise sources do not substantially alter the results and conclusions of this paper.

Synthetic recovery tests also support the detected high and low velocity anomalies. We set up two input 3D velocity models: One contained synthetic low and high velocity anomalies at 5- to 16-km and 5- to 10-km depths, respectively (fig. S13); the other used our final 3D velocity model estimated from the real data as the input model (fig. S14). We computed synthetic travel time data using the finite-frequency kernels and added Gaussian noise whose standard deviations were the same as those of the pairwise phase velocity measurements from the real data. The phase velocity tomography and subsequent 1D inversions were conducted with the same regularization parameters used for the real data. The output models show the low and high velocity anomalies of the input models, indicating that the structural feature we discuss is resolved from the dataset and tomographic inversion (figs. S13 and S14).

#### Comparison with other 3D structure models

We compared our *S*-wave velocity structure with other 3D velocity models estimated by body-wave tomography using permanent seismic networks (fig. S15) (*12*, *18*). Our results have a much higher resolution owing to the dense seismic networks in the northeastern Noto Peninsula. The previous models do not show the structural features discussed in this study because of the limited resolution of the permanent seismic networks.

## Supplementary Materials

### This PDF file includes:

Figs. S1 to S15 References
