# Supplementary material for: Rupture of solidified ancient magma that impeded preceding swarm migrations led to the 2024 Noto earthquake
Source: Sci Adv. 2025 Oct 15;11(42):eadv5938. doi: 10.1126/sciadv.adv5938 (PMC12526457; doi:10.1126/sciadv.adv5938)
Supplement: Supplementary file 1 — Figs. S1 to S15 References [file sciadv.adv5938_sm.pdf]

Science Advances
AAAS

Supplementary Materials for

Rupture of solidified ancient magma that impeded preceding swarm migrations led to the 2024 Noto earthquake

Ryota Takagi *et al.*

Corresponding author: Ryota Takagi, ryota.takagi.c1@tohoku.ac.jp

Sci. Adv. **11**, eadv5938 (2025)
DOI: 10.1126/sciadv.adv5938

This PDF file includes:

Figs. S1 to S15
References

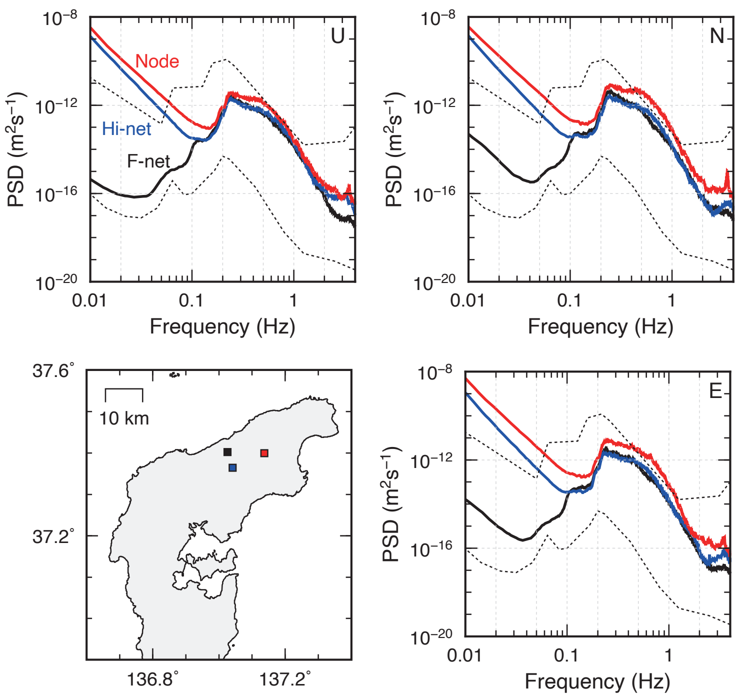

Fig. S1. Power spectral density of a node, Hi-net, and F-net stations. The median power spectral densities of an IGU-BD3C-5 seismic node (red), Hi-net (blue), and F-net (black) stations for two-week period from November 14 to 28, 2023, are shown. The dashed curves are the new low and high noise models (*54*).

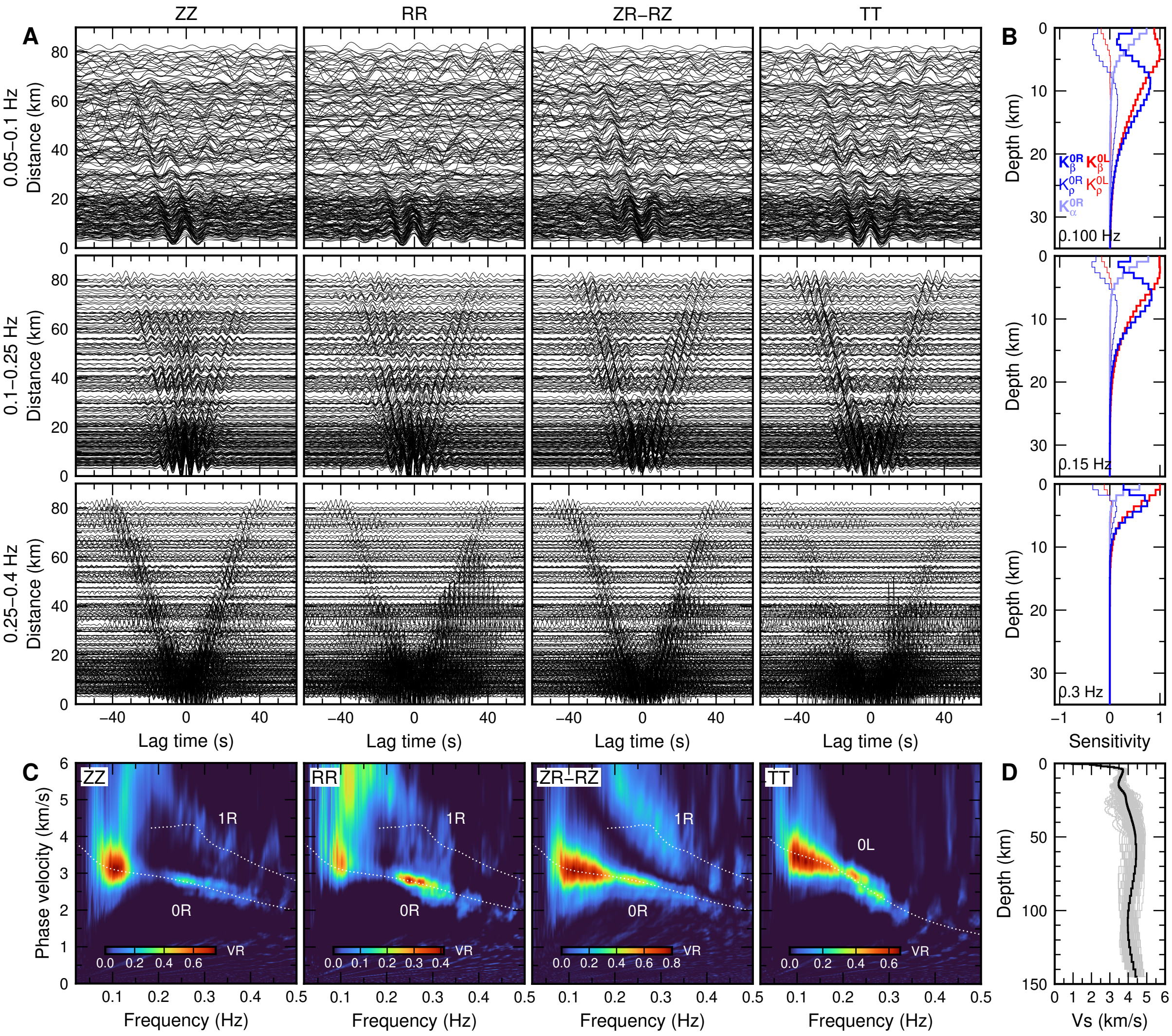

Fig. S2. Surface waves extracted by cross-correlation functions of ambient seismic noise. **(A)** Multicomponent cross-correlation functions for three frequency bands aligned with respect to interstation distance. The positive and negative lag times correspond to the eastward and westward propagations, respectively. **(B)** Normalized sensitivity kernels for the fundamental mode Rayleigh (blue) and Love waves (red). The thick curves are the sensitivity to S-wave velocity, thin curves to density, and light-colored curve is to P-wave velocity. **(C)** Dispersion spectra shown by the variance reduction of waveform fitting using all seismic stations. The dotted curves are the fundamental-mode (0R) and first-overtone Rayleigh waves (1R), and the fundamental-mode Love wave (0L) calculated by the 1D structure model shown in (D). **(D)** The gray curves indicate the 1D S-wave velocity models from 100 bootstrap resampled data and the black curve is the average of them.

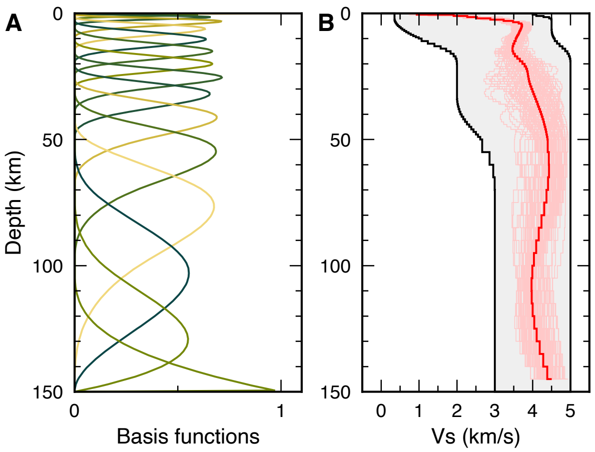

Fig. S3. Model parameters for the dispersion measurement. (A) Cubic B-spline functions representing the 1D Vs structure model. **(B)** The searching range of the reference 1D Vs structure in the first step. The thin and thick red curves are the estimated results from 100 bootstrap samples and their mean.

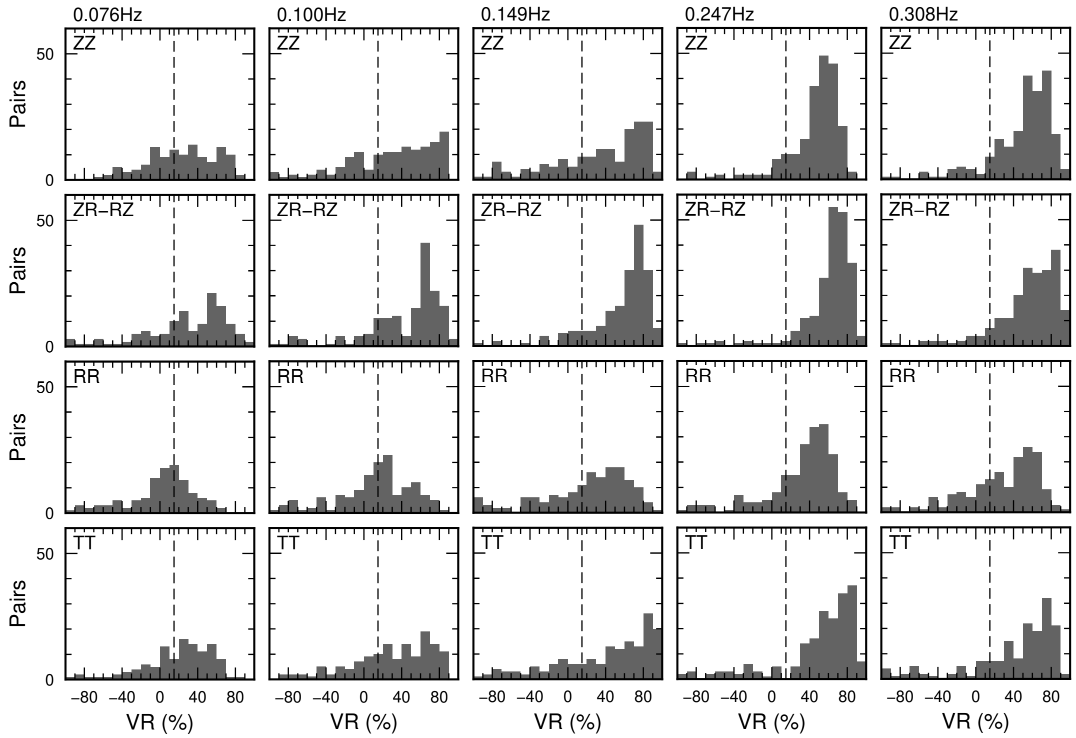

Fig. S4. Variance reduction of the pairwise cross-spectral fitting for phase velocity measurements at each station pair. Station pairs with separations longer than half a wavelength are shown. The vertical dashed line represents the threshold value we set.

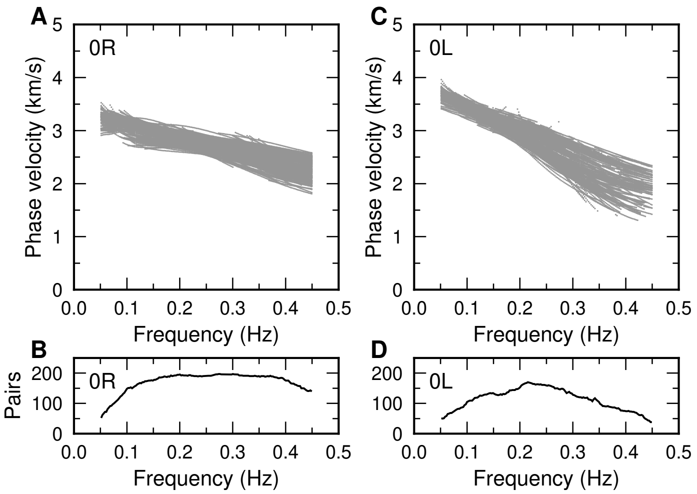

Fig. S5. Phase velocity dispersion curves after the quality control. (A) Measured dispersion curves for the fundamental-mode Rayleigh waves after the quality control. **(B)** The number of station pairs for the Rayleigh wave measurements after the quality control. **(C, D)** The fundamental-mode Love waves.

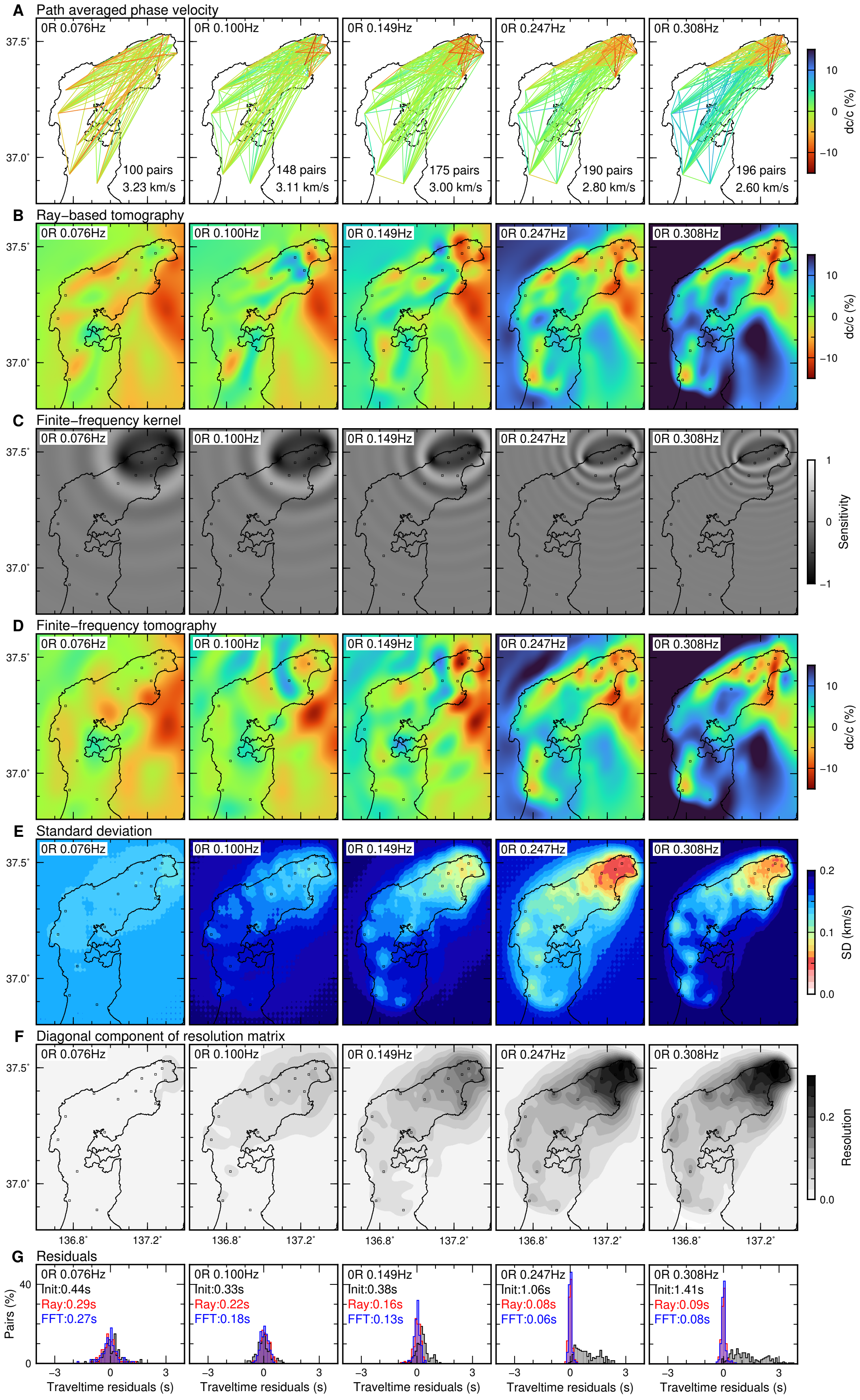
A Path averaged phase velocity
0R 0.076Hz
0R 0.100Hz
0R 0.149Hz
0R 0.247Hz
0R 0.308Hz
100 pairs
3.23 km/s
148 pairs
3.11 km/s
175 pairs
3.00 km/s
190 pairs
2.80 km/s
196 pairs
2.60 km/s
dc/c (%)
B Ray−based tomography
C Finite−frequency kernel
Sensitivity
D Finite−frequency tomography
E Standard deviation
SD (km/s)
F Diagonal component of resolution matrix
Resolution
G Residuals
Init:0.44s
Ray:0.29s
FFT:0.27s
Init:0.33s
Ray:0.22s
FFT:0.18s
Init:0.38s
Ray:0.16s
FFT:0.13s
Init:1.06s
Ray:0.08s
FFT:0.06s
Init:1.41s
Ray:0.09s
FFT:0.08s
Pairs (%)
Traveltime residuals (s)
37.5˚
37.0˚
136.8˚
137.2˚

Fig. S6. Phase velocity tomography for Rayleigh waves. The reference velocities for the phase velocity perturbations are the array-based phase velocity estimates (Fig. S2C). **(A)** The path-averaged phase velocity. **(B)** Ray-based tomography. **(C)** The finite-frequency kernels. The amplitude is normalized by the maximum value. **(D)** Finite-frequency tomography. **(E)** Standard deviation (the diagonal component of posterior covariance matrix) of the finite-frequency tomography results. **(F)** The diagonal components of the resolution matrix of the finite-frequency tomography. **(G)** Travel time residuals for the initial phase velocity maps (gray), ray-based tomography (red), and finite-frequency tomography (blue).

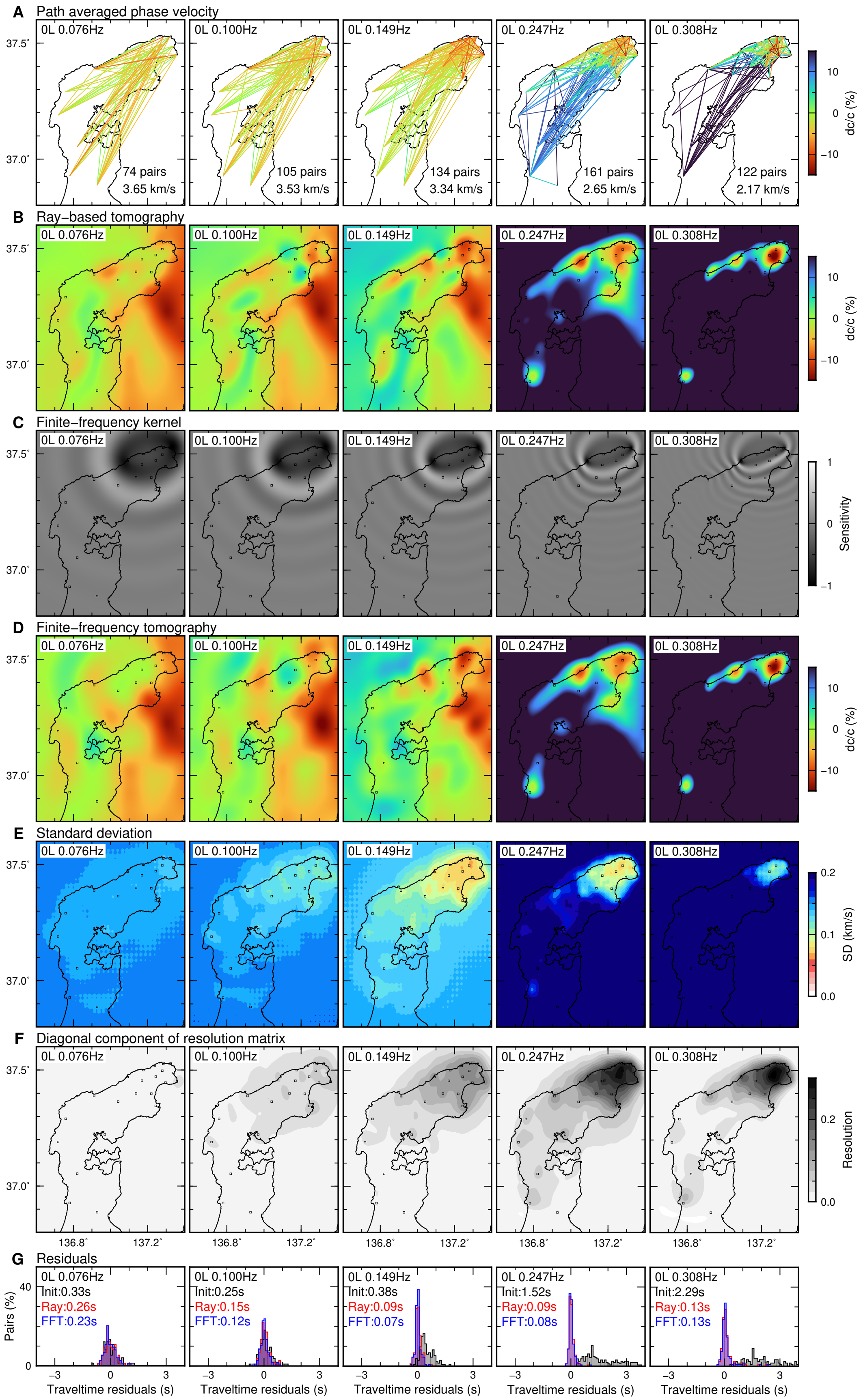

A Path averaged phase velocity
0L 0.076Hz
74 pairs
3.65 km/s
0L 0.100Hz
105 pairs
3.53 km/s
0L 0.149Hz
134 pairs
3.34 km/s
0L 0.247Hz
161 pairs
2.65 km/s
0L 0.308Hz
122 pairs
2.17 km/s
dc/c (%)
B Ray−based tomography
C Finite−frequency kernel
Sensitivity
D Finite−frequency tomography
E Standard deviation
SD (km/s)
F Diagonal component of resolution matrix
Resolution
37.5˚
37.0˚
136.8˚
137.2˚
G Residuals
Pairs (%)
0L 0.076Hz
Init:0.33s
Ray:0.26s
FFT:0.23s
0L 0.100Hz
Init:0.25s
Ray:0.15s
FFT:0.12s
0L 0.149Hz
Init:0.38s
Ray:0.09s
FFT:0.07s
0L 0.247Hz
Init:1.52s
Ray:0.09s
FFT:0.08s
0L 0.308Hz
Init:2.29s
Ray:0.13s
FFT:0.13s
Traveltime residuals (s)

Fig. S7. Phase velocity tomography for Love waves. The reference velocities for the phase velocity perturbations are the array-based phase velocity estimates (Fig. S2C). **(A)** The path-averaged phase velocity. **(B)** Ray-based tomography. **(C)** The finite-frequency kernels. The amplitude is normalized by the maximum value. **(D)** Finite-frequency tomography. **(E)** Standard deviation (the diagonal component of posterior covariance matrix) of the finite-frequency tomography results. **(F)** The diagonal components of the resolution matrix of the finite-frequency tomography. **(G)** Travel time residuals for the initial phase velocity maps (gray), ray-based tomography (red), and finite-frequency tomography (blue).

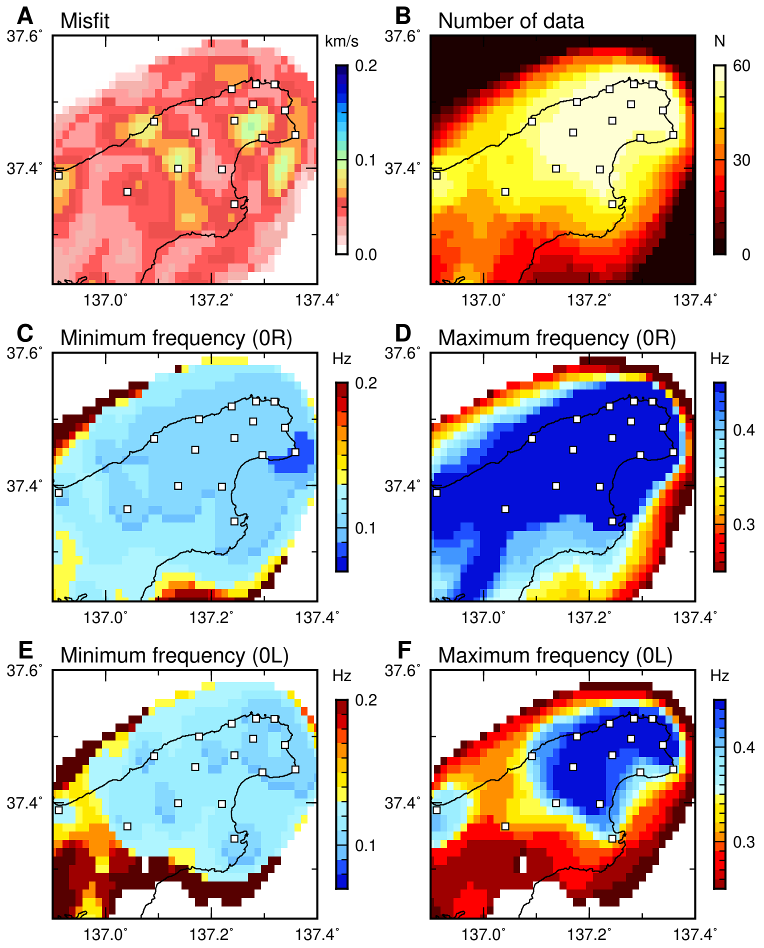

Fig. S8. Misfit values of the 1D inversions and the number and frequency ranges used in the inversions. (A) The root mean square residuals of the phase velocity data at each grid point. **(B)** The number of data used in the 1D inversion at each grid point. **(C)** The minimum frequency of the Rayleigh-wave phase velocity data. **(D)** The maximum frequency of the Rayleigh-wave phase velocity data. **(E, F)** The frequency range of the Love-wave data.

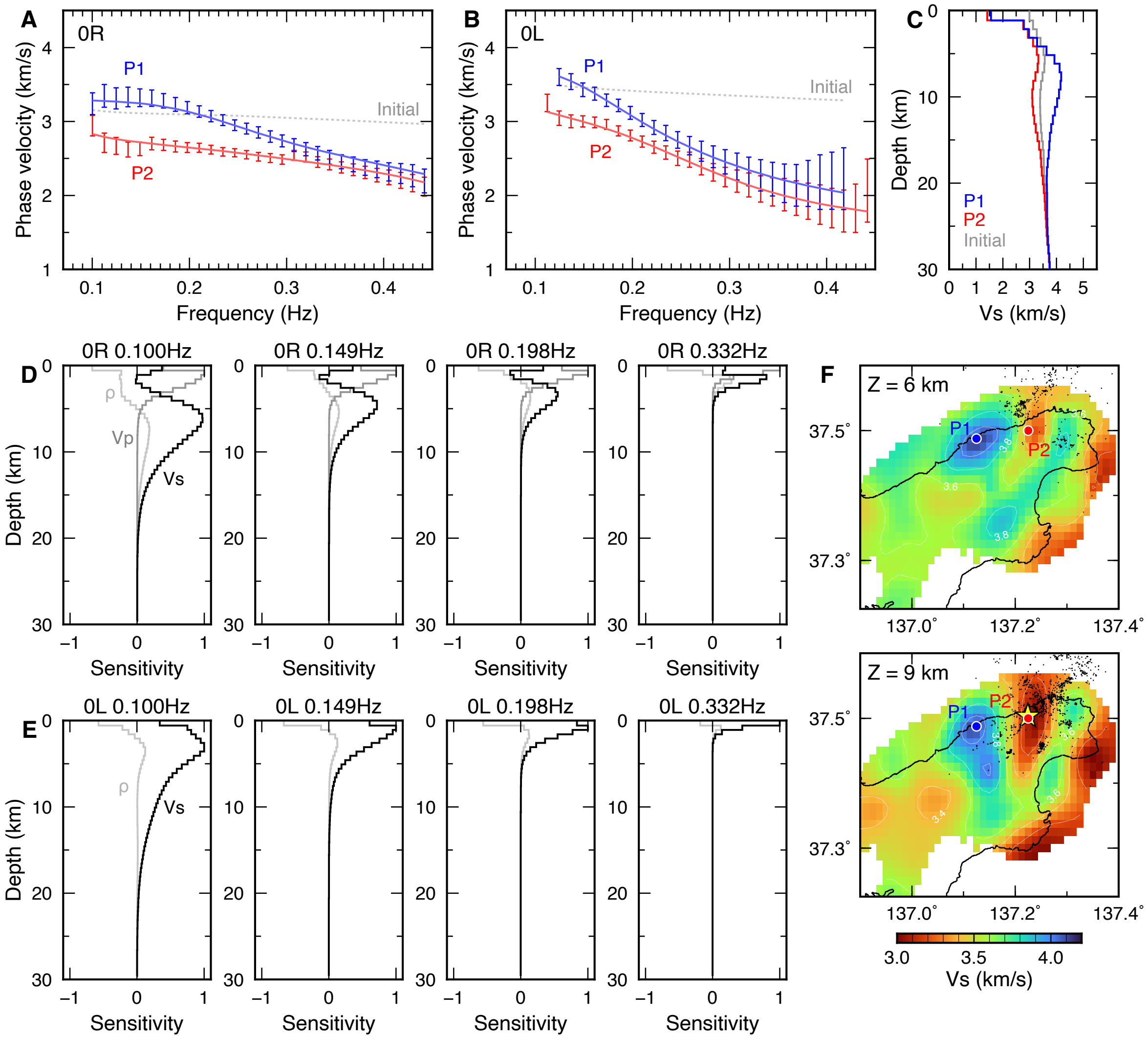

Fig. S9. Examples of the local 1D inversion. The Rayleigh and Love waves phase velocities are inverted simultaneously with the assumption of isotropic Vs. **(A)** The Rayleigh-wave local phase velocity data (1σ error bars) and the dispersion curves computed from the final velocity models (solid curves) at the two locations, P1 (blue) and P2 (red). The gray curve represents the initial dispersion curve at P1. **(B)** The Love-wave local phase velocity data and dispersion curves. **(C)** The initial and final Vs models. **(D)** Sensitivity kernels of the fundamental-mode Rayleigh waves computed with the final velocity model at P1. **(E)** Sensitivity kernels of the fundamental-mode Love waves. **(F)** Map showing locations of P1 and P2.

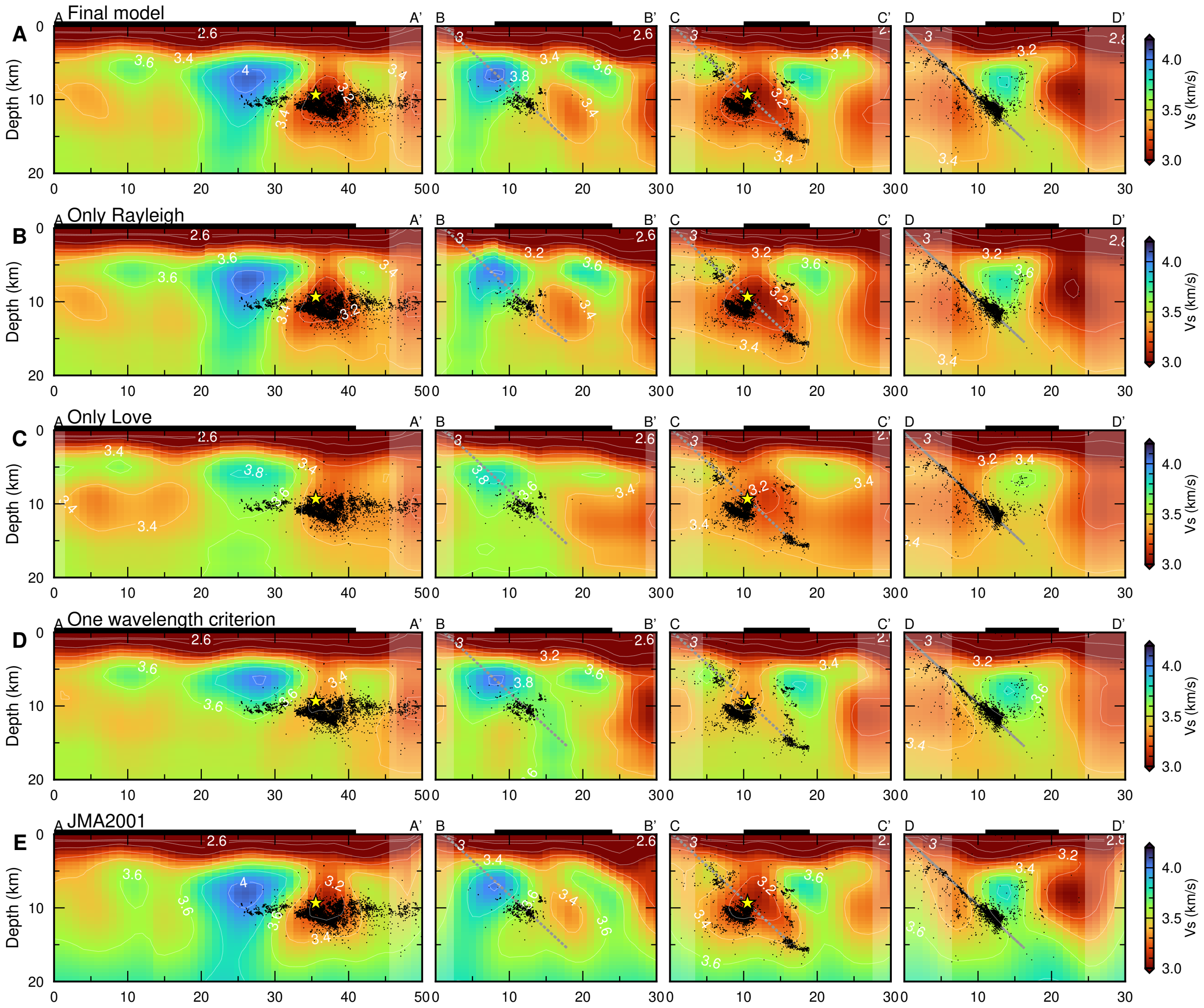

Fig. S10. 3D S-wave velocity structures estimated with different data selections or different initial model. The profiles are the same as Fig. 3. **(A)** The final Vs model of the present study estimated with both Rayleigh and Love waves, the half wavelength criterion, and the initial 3D model (*43*). **(B)** The Vs model estimated from only Rayleigh waves. **(C)** The Vs model estimated from only Love waves. **(D)** The Vs model estimated from only phase velocity measurements with station separations longer than one wavelength. **(E)** The Vs model estimated using the JMA2001 1D velocity structure as the initial model. The JMA2001 model is used for the hypocenter location by JMA (*55*).

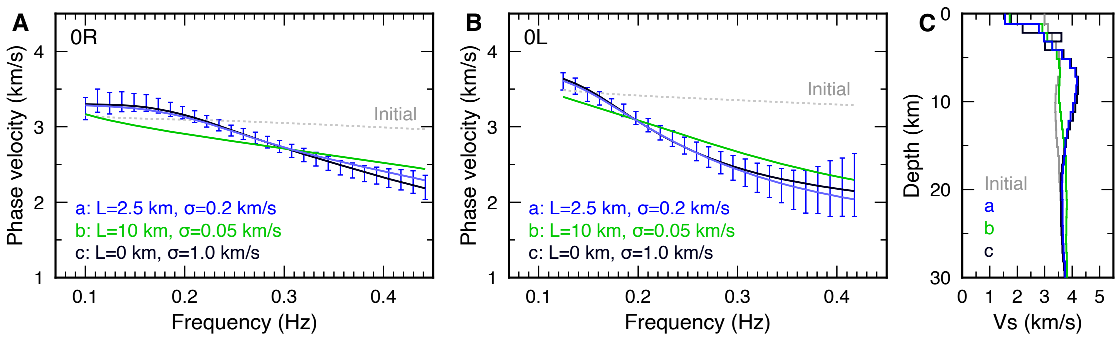

Fig. S11. Dependence on a priori model covariance matrix in the 1D inversion. The results at P1 (fig. S7F) area shown. **(A)** The Rayleigh wave data and dispersion curves. The blue, green, and navy curves represent the inversion results with different correlation lengths (L) and standard deviations (σ). **(B)** The Love wave data and dispersion curves. **(C)** The initial and final Vs models estimated with the three parameter sets.

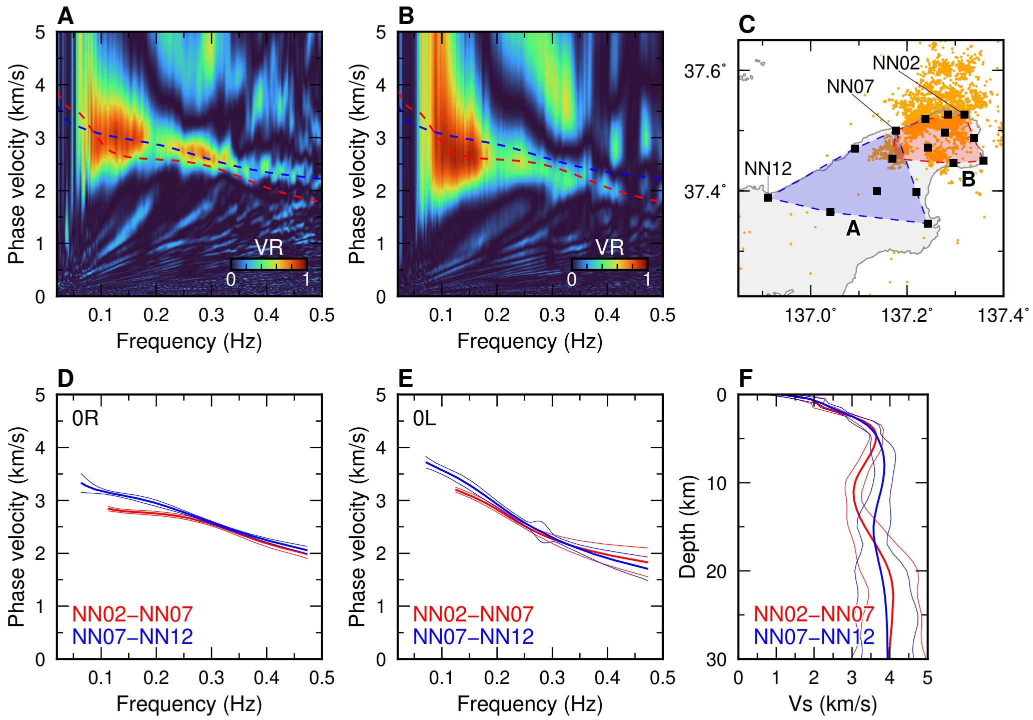

Fig. S12. Phase velocity measurements in the swarm area and its adjacent western region. **(A)** Array-based phase velocity measurement in the western region (region A). The color map shows the variance reduction of the cross-spectral fitting for the ZR–RZ component and the dashed curves shows the measured dispersion curves of the fundamental-mode Rayleigh waves (blue: region A, red: region B). **(B)** Array-based phase velocity measurement in the swarm area (region B). **(C)** Map showing subarrays and station pairs. **(D)** Phase velocity measurements of the fundamental-mode Rayleigh waves for two individual station pairs: NN02–NN07 (red) and NN07–NN12 (blue). **(E)** Phase velocity measurements of the fundamental-mode Love waves for the two station pairs. **(F)** The 1D Vs models, the results of the pairwise cross-spectral fitting, for the two station pairs.

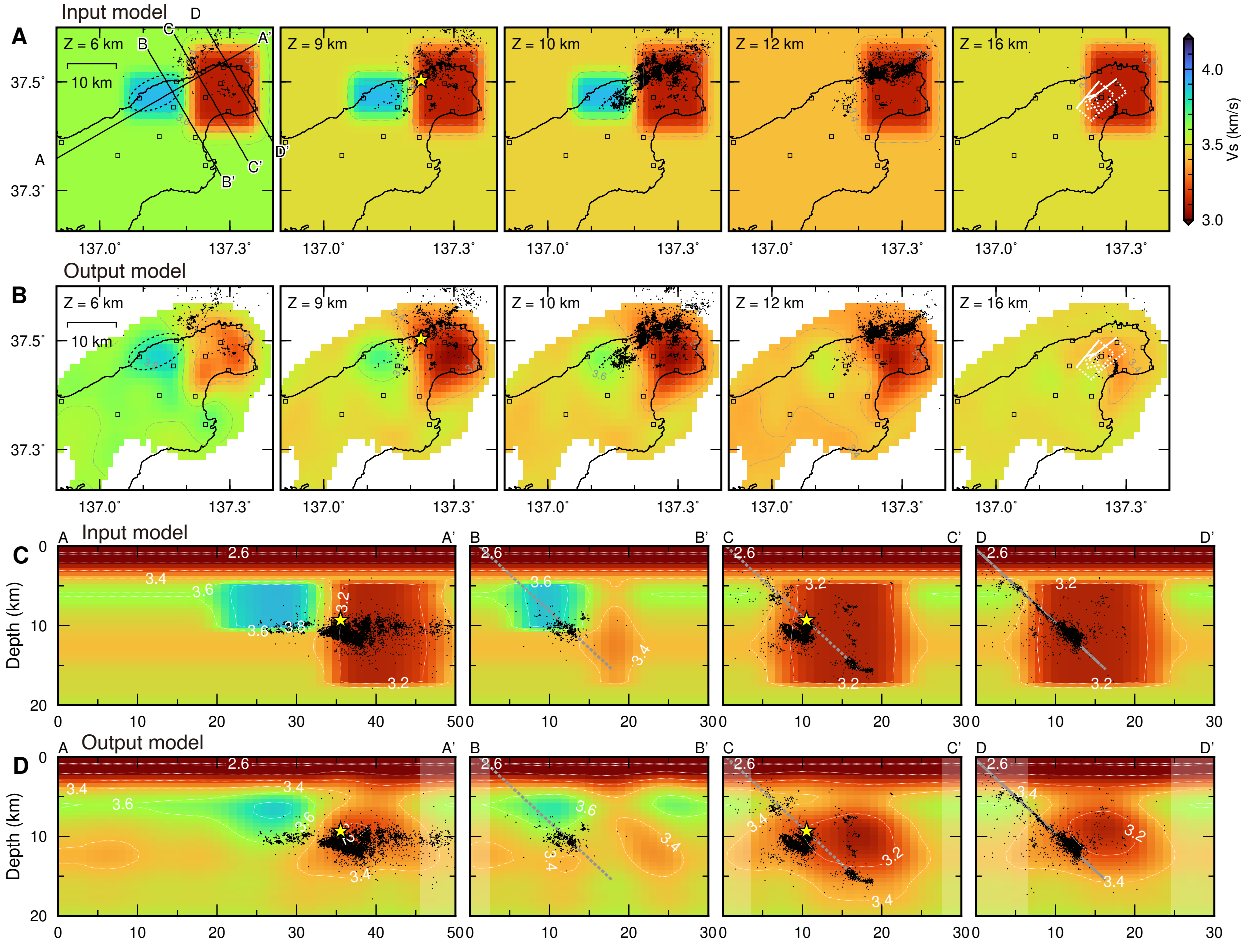

Fig. S13. Synthetic test with distinct low and high velocity anomalies. (A) Horizontal cross sections of the input model. **(B)** Horizontal cross sections of the output model. **(C)** Vertical cross sections of the input model. **(D)** Vertical cross sections of the output model. The symbols are the same as Figs. 2 and 3.

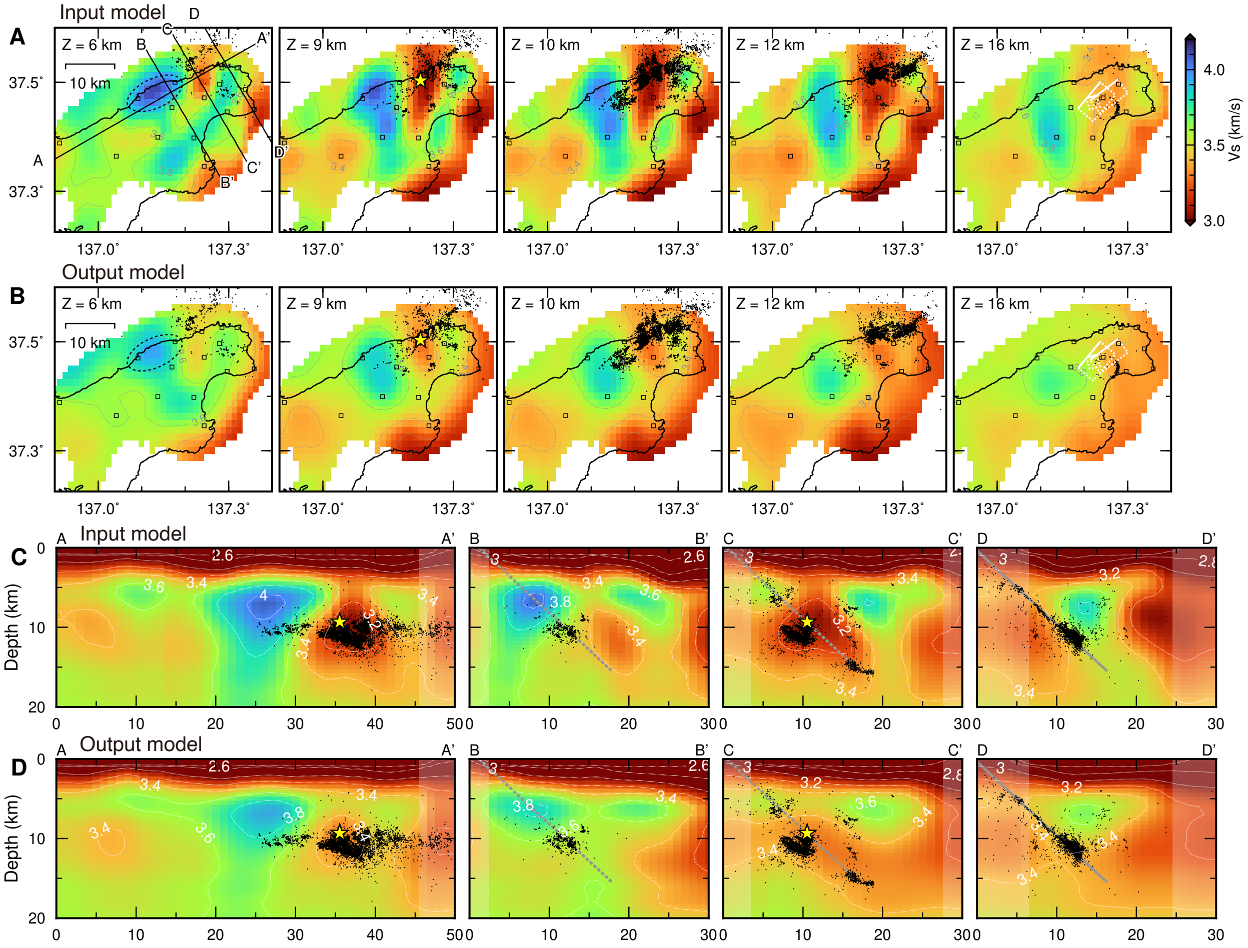

Fig. S14. Synthetic test using the final model as the input model. (A) Horizontal cross sections of the input model. **(B)** Horizontal cross sections of the output model. **(C)** Vertical cross sections of the input model. **(D)** Vertical cross sections of the output model. The symbols are the same as Figs. 2 and 3.

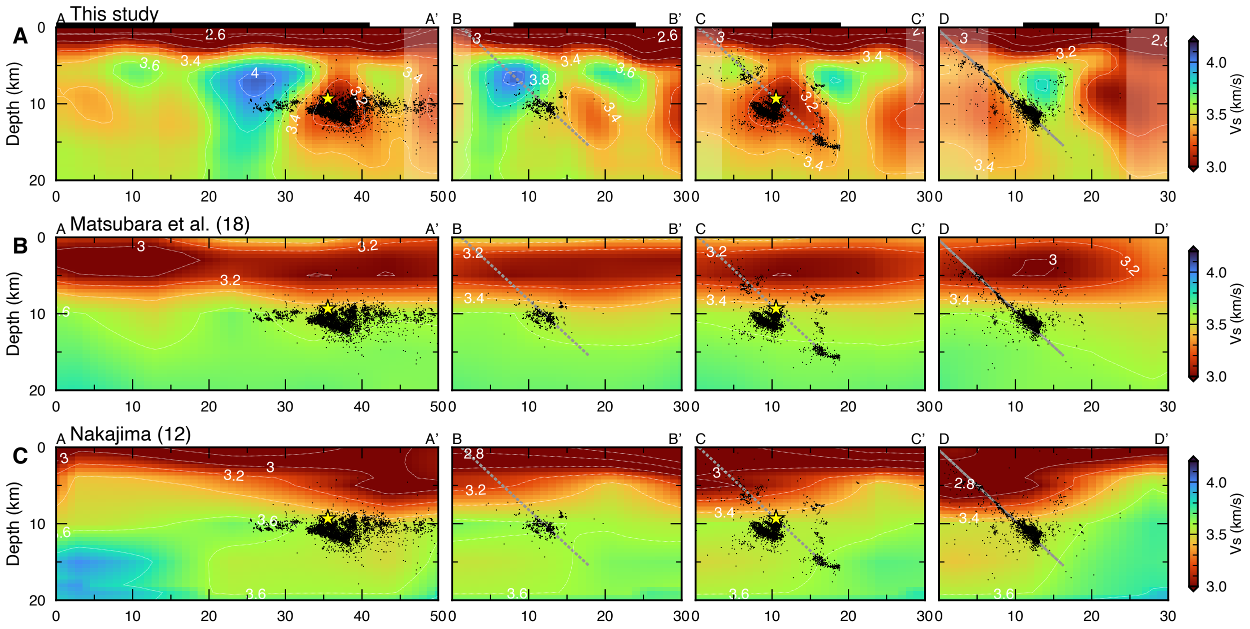

Fig. S15. Comparison with other structure models. S-wave velocity models estimated in the present study and previous works (*12, 18*) are compared in the same scale. The profiles are the same as Fig. 3.

REFERENCES AND NOTES

1. M. Hamada, Y. Hiramatsu, M. Oda, H. Yamaguchi, Fossil tubeworms link coastal uplift of the northern Noto Peninsula to rupture of the Wajima-oki fault in AD 1729. *Tectonophysics* **670**, 38–47 (2016).

2. R. Okuwaki, Y. Yagi, A. Murakami, Y. Fukahata, A multiplex rupture sequence under complex fault network due to preceding earthquake swarms during the 2024 Mw 7.5 Noto Peninsula, Japan, earthquake. *Geophys. Res. Lett.* **51**, e2024GL109224 (2024).

3. Z. Ma, H. Zeng, H. Luo, Z. Liu, Y. Jiang, Y. Aoki, W. Wang, Y. Itoh, M. Lyu, Y. Cui, S. H. Yun, E. M. Hill, S. Wei, Slow rupture in a fluid-rich fault zone initiated the 2024 Mw 7.5 Noto earthquake. *Science* **385**, 866–871 (2024).

4. M. Shishikura, T. Echigo, Y. Namegaya, Activity of the off-shore active faults along the northern coast of the Noto Peninsula deduced from the height distribution of the lower marine terrace and emerged sessile assemblage. *Active Fault Res.* **2020**, 33–49 (2020).

5. Y. Fukushima, D. Ishimura, N. Takahashi, Y. Iwasa, L. C. Malatesta, T. Takahashi, C. H. Tang, K. Yoshida, S. Toda, Landscape changes caused by the 2024 Noto Peninsula earthquake in Japan. *Sci. Adv.* **10**, eadp9193 (2024).

6. M. Ozaki, “1:200,000 Geological map of the northern part of Noto Peninsula” in *Seamless Geoinformation of Coastal Zone “Northern Coastal Zone of Noto Peninsula”* (S-1, Geological Survey of Japan, AIST, 2010).

7. Y. Amezawa, Y. Hiramatsu, A. Miyakawa, K. Imanishi, M. Otsubo, Long-living earthquake swarm and intermittent seismicity in the northeastern tip of the Noto Peninsula, Japan. *Geophys. Res. Lett.* **50**, e2022GL102670 (2023).

8. K. Yoshida, N. Uchida, Y. Matsumoto, M. Orimo, T. Okada, S. Hirahara, S. Kimura, R. Hino, “Earthquake data for the 2023 Mw6.2 Suzu Earthquake in the Northeastern Noto Peninsula, Japan” (2023); https://doi.org/10.5281/zenodo.10019860.

9. K. Yoshida, M. Uno, T. Matsuzawa, Y. Yukutake, Y. Mukuhira, H. Sato, T. Yoshida, Upward earthquake swarm migration in the northeastern Noto Peninsula, Japan, initiated from a deep ring-shaped cluster: Possibility of fluid leakage from a hidden magma system. *J. Geophys. Res. Solid Earth* **128**, e2022JB026047 (2023).

10. A. Kato, Implications of fault-valve behavior from immediate aftershocks following the 2023 Mj 6.5 earthquake beneath the Noto Peninsula, central Japan. *Geophys. Res. Lett.* **51**, e2023GL106444 (2024).

11. T. Nishimura, Y. Hiramatsu, Y. Ohta, Episodic transient deformation revealed by the analysis of multiple GNSS networks in the Noto Peninsula, central Japan. *Sci. Rep.* **13**, 8381 (2023).

12. J. Nakajima, Crustal structure beneath earthquake swarm in the Noto peninsula, Japan. *Earth Planets Space* **74**, 160 (2022).

13. T. Okada, M. K. Savage, S. Sakai, K. Yoshida, N. Uchida, R. Takagi, S. Kimura, S. Hirahara, A. Tagami, R. Fujimura, T. Matsuzawa, E. Kurashimo, Y. Hiramatsu, Shear wave splitting and seismic velocity structure in the focal area of the earthquake swarm and their relation with earthquake swarm activity in the Noto Peninsula, central Japan. *Earth Planets Space* **76**, 24 (2024).

14. R. Yoshimura, Y. Hiramatsu, T. Goto, T. Inui, M. Yoshikawa, A. Namigishi, A. Nagaoka, J. Nakatawa, R. Miyamachi, A. Sawada, M. Fukada, A. Suii, C. Zhang, N. Yamashita, Y. Oshima, M. Kanazawa, R. Amano, "Three-dimensional electrical resistivity structure around earthquake swarm region in the northeastern Noto Peninsula," in *152th Society of Geomagnetism and Earth, Planetary and Space Sciences* (Society of Geomagnetism and Earth, Planetary and Space Sciences, 2022).

15. J. Mori, C. McKee, Outward-dipping ring-fault structure at Rabaul caldera as shown by earthquake locations. *Science* **235**, 193–195 (1987).

16. L. Xu, C. Ji, L. Meng, J. P. Ampuero, Z. Yunjun, S. Mohanna, Y. Aoki, Dual-initiation ruptures in the 2024 Noto earthquake encircling a fault asperity at a swarm edge. *Science* **385**, 871–876 (2024).

17. K. Yoshida, R. Takagi, Y. Fukushima, R. Ando, Y. Ohta, Y. Hiramatsu, Role of a hidden fault in the early process of the 2024 Mw7.5 Noto Peninsula earthquake. *Geophys. Res. Lett.* **51**, e2024GL110993 (2024).

18. M. Matsubara, T. Ishiyama, T. No, K. Uehira, M. Mochizuki, T. Kanazawa, N. Takahashi, S. Kamiya, Seismic velocity structure along the Sea of Japan with large events derived from seismic tomography for whole Japanese Islands including reflection survey data and NIED MOWLAS Hi-net and S-net data. *Earth Planets Space* **74**, 171 (2022).

19. National Research Institute for Earth Science and Disaster Resilience, "NIED Hi-net" (2019); https://doi.org/10.17598/NIED.0003.

20. N. M. Shapiro, M. Campillo, L. Stehly, M. H. Ritzwoller, High-resolution surface-wave tomography from ambient seismic noise. *Science* **307**, 1615–1618 (2005).

21. Geological Survey of Japan, AIST, "Seamless digital geological map of Japan V2 1: 200,000, original edition" (2025); https://gbank.gsj.jp/seamless/.

22. T. Yoshikawa, K. Kano, Y. Yanagisawa, M. Komazawa, M. Joshima, E. Kikawa, "Geology of the Suzumisaki, Noto-iida and Horyu-zan district" in *Quadrangle Series, 1:50,000* (Geological Survey of Japan, AIST, 2002).

23. N. I. Christensen, Poisson's ratio and crustal seismology. *J. Geophys. Res. Solid Earth* **101**, 3139–3156 (1996).

24. P. B. Kelemen, W. S. Holbrook, Origin of thick, high-velocity igneous crust along the U.S. East Coast Margin. *J. Geophys. Res. Solid Earth* **100**, 10077–10094 (1995).

25. S. E. Johnson, K. L. Schmidt, M. C. Tate, Ring complexes in the Peninsular Ranges Batholith, Mexico and the USA: Magma plumbing systems in the middle and upper crust. *Lithos.* **61**, 187–208 (2002).

26. R. H. Sibson, Implications of fault-valve behaviour for rupture nucleation and recurrence. *Tectonophysics* **211**, 283–293 (1992).

27. D. R. Faulkner, C. A. L. Jackson, R. J. Lunn, R. W. Schlische, Z. K. Shipton, C. A. J. Wibberley, M. O. Withjack, A review of recent developments concerning the structure, mechanics and fluid flow properties of fault zones. *J. Struct. Geol.* **32**, 1557–1575 (2010).

28. R. T. Williams, Å. Fagereng, The role of quartz cementation in the seismic cycle: A critical review. *Rev. Geophys.* **60**, e2021RG000768 (2022).

29. H. Guo, T. Lay, E. E. Brodsky, Seismological indicators of geologically inferred fault maturity. *J. Geophys. Res. Solid Earth* **128**, e2023JB027096 (2023).

30. P. Thakur, Y. Huang, Influence of fault zone maturity on fully dynamic earthquake cycles. *Geophys. Res. Lett.* **48**, e2021GL094679 (2021).

31. Z. E. Ross, E. S. Cochran, D. T. Trugman, J. D. Smith, 3D fault architecture controls the dynamism of earthquake swarms. *Science* **368**, 1357–1361 (2020).

32. D. R. Shelly, D. P. Hill, F. Massin, J. Farrell, R. B. Smith, T. Taira, A fluid-driven earthquake swarm on the margin of the Yellowstone caldera. *J. Geophys. Res. Solid Earth* **118**, 4872–4886 (2013).

33. Y. Yukutake, H. Ito, R. Honda, M. Harada, T. Tanada, A. Yoshida, Fluid-induced swarm earthquake sequence revealed by precisely determined hypocenters and focal mechanisms in the 2009 activity at Hakone volcano. *Japan. J. Geophys. Res. Solid Earth* **116**, 4308 (2011).

34. R. Takagi, G. Toyokuni, N. Chikasada, Ambient noise correlation analysis of S-net records: Extracting surface wave signals below instrument noise levels. *Geophys. J. Int.* **224**, 1640–1657 (2021).

35. K. Nishida, R. Takagi, A global centroid single force catalog of P-wave microseisms. *J. Geophys. Res. Solid Earth* **127**, e2021JB023484 (2022).

36. R. Takagi, H. Nakahara, T. Kono, T. Okada, Separating body and Rayleigh waves with cross terms of the cross-correlation tensor of ambient noise. *J. Geophys. Res. Solid Earth* **119**, 2005–2018 (2014).

37. K. Nishida, R. Takagi, A. Takeo, Ambient noise multimode surface wave tomography. *Prog. Earth Planet Sci. 11*, 4 (2024).

38. R. Takagi, K. Nishida, Multimode dispersion measurement of surface waves extracted by multicomponent ambient noise cross-correlation functions. *Geophys. J. Int.* **231**, 1196–1220 (2022).

39. Z. Li, C. Shi, X. Chen, Constraints on crustal P wave structure with leaking mode dispersion curves. *Geophys. Res. Lett.* **48**, e2020GL091782 (2021).

40. N. Rawlinson, M. Sambridge, The fast marching method: An effective tool for tomographic imaging and tracking multiple phases in complex layered media. *Explor. Geophys.* **36**, 341–350 (2005).

41. K. Yoshizawa, B. L. N. Kennett, Sensitivity kernels for finite-frequency surface waves. *Geophys J Int* **162**, 910–926 (2005).

42. K. Yoshizawa, B. L. N. Kennett, Multimode surface wave tomography for the Australian region using a three-stage approach incorporating finite frequency effects. *J. Geophys. Res. Solid Earth* **109**, B02310 (2004).

43. K. Nishida, H. Kawakatsu, K. Obara, Three-dimensional crustal S wave velocity structure in Japan using microseismic data recorded by Hi-net tiltmeters. *J. Geophys. Res. Solid Earth* **113**, B10302 (2008).

44. Y. Mitsuhata, T. Uchida, H. Amano, 2.5-D inversion of frequency-domain electromagnetic data generated by a grounded-wire source. *Geophysics* **67**, 1753–1768 (2002).

45. M. Matsu'ura, A. Noda, Y. Fukahata, Geodetic data inversion based on Bayesian formulation with direct and indirect prior information. *Geophys. J. Int.* **171**, 1342–1351 (2007).

46. A. Tarantola, B. Valette, Generalized nonlinear inverse problems solved using the least squares criterion. *Rev. Geophys.* **20**, 219–232 (1982).

47. C. E. Nishimura, D. W. Forsyth, The anisotropic structure of the upper mantle in the Pacific. *Geophys. J. Int.* **96**, 203–229 (1989).

48. M. Saito, "DISPER80: A subroutine package for the calculation of seismic normal mode solutions" in *Seismological Algorithms: Computational Methods and Computer Programs* (Academic Press, 1988), pp. 293–319.

49. T. M. Brocher, Empirical relations between elastic wavespeeds and density in the Earth's crust. *Bull. Seismol. Soc. Am.* **95**, 2081–2092 (2005).

50. R. Weaver, B. Froment, M. Campillo, On the correlation of non-isotropically distributed ballistic scalar diffuse waves. *J. Acoust. Soc. Am.* **126**, 1817–1826 (2009).

51. G. Ekström, M. Nettles, A. M. Dziewoński, The global CMT project 2004–2010: Centroid-moment tensors for 13,017 earthquakes. *Phys. Earth Planet. Inter.* **200–201**, 1–9 (2012).

52. A. Kubo, E. Fukuyama, H. Kawai, K. Nonomura, NIED seismic moment tensor catalogue for regional earthquakes around Japan: Quality test and application. *Tectonophysics* **356**, 23–48 (2002).

53. National Institute of Advanced Industrial Science and Technology, "Active Fault Database of Japan, February 28, 2012 version" (2012); https://gbank.gsj.jp/activefault/index_e_gmap.html.

54. J. R. Peterson, "Observations and modeling of seismic background noise" (Open File Report Series Number 93-322, U.S. Geological Survey, 1993), 94 p.

55. H. Ueno, S. Hatakeyam, T. Aketagawa, J. Funasaki, N. Hamada, Improvement of hypocenter determination procedures in the Japan Meteorological Agency. *Quart. J. Seismol.* **65**, 123–134 (2002).
